# Gastric Cancer-Derived Exosomes Activate Mast Cells via the SCF/c-KIT Pathway to Drive Angiogenesis and Metastasis

**DOI:** 10.32604/or.2026.085030

**Published:** 2026-09-14

**Authors:** Shaoxiong Bai, Yilei Duan, Tian Yao, Yanan Shi, Xin Zhang, Xiaole Ma, Kai Jia

**Affiliations:** 1 Department of Gastrointestinal Surgery, First Hospital of Shanxi Medical University, Taiyuan, China; 2 Medical Department, First Hospital of Shanxi Medical University, Taiyuan, China; 3 Department of Gastrointestinal Surgery, Shanxi Cancer Hospital, Taiyuan, China

**Keywords:** Gastric cancer, exosome, mast cell, angiogenesis, cancer metastasis

## Abstract

**Background:** Exosomes mediate intercellular communication within the tumor microenvironment. However, their role in modulating mast cell activity in gastric cancer (GC) remains unclear. This study aimed to elucidate whether GC-derived exosomes activate mast cells via the SCF/c-KIT pathway to promote angiogenesis and metastasis, and to assess the therapeutic potential of targeting this axis. **Methods:** Exosomes were isolated from GC cell lines (AGS, MKN1), a normal gastric epithelial cell line (GES-1), and mouse gastric tumor tissues, followed by characterization via NTA, TEM, and western blot. Mast cell (LAD2) degranulation was quantified by β-hexosaminidase release and ELISA. Cell recruitment was evaluated using Transwell migration assays, and angiogenesis was assessed by tube formation assay. Underlying signaling pathways were analyzed by Western blot. The functional role of the SCF/c-KIT axis was investigated using shRNA-mediated knockdown and neutralizing antibodies. A mouse model of gastric cancer lung metastasis was established to validate the *in vivo* effects. **Results:** GC-derived exosomes, but not GES-1-derived ones, were enriched with SCF and induced mast cell recruitment, degranulation, and tryptase release via SCF/c-KIT activation. Mast cell-derived tryptase promoted angiogenesis by activating the MAPK pathway and upregulating VEGF in endothelial cells. *In vivo*, tumor-derived exosomes accelerated lung metastasis and angiogenesis, effects abrogated by a c-KIT blocking antibody, confirming SCF/c-KIT dependence. **Conclusion:** Gastric cancer-derived exosomes deliver SCF to activate mast cells via the c-KIT receptor, thereby fostering angiogenesis and metastasis. Targeting the exosomal SCF/c-KIT signaling axis may offer a promising therapeutic strategy to impede gastric cancer progression.

## 1 Introduction

Gastric cancer (GC) continues to pose a substantial global health challenge. It is currently ranked as the fifth most frequently diagnosed malignancy and the fourth leading cause of cancer-associated deaths worldwide [1]. Although significant progress has been made in standard treatment modalities, including refined surgical resection and the development of novel adjuvant chemotherapeutic regimens, the clinical outcomes for patients with advanced-stage or recurrent disease remain unsatisfactory. The overall prognosis is particularly dismal, with a five-year survival rate persistently below 30% for these patient groups [2]. The high mortality rate is predominantly attributed to distinct malignant behaviors, specifically distant metastasis and uncontrolled angiogenesis [3]. Therefore, elucidating the molecular mechanisms governing the interplay between GC cells and the surrounding stromal microenvironment is crucial for identifying novel therapeutic targets to inhibit disease progression.

The tumor microenvironment (TME) constitutes a complex ecosystem where cancer cells coexist and interact with various stromal components, including immune cells, fibroblasts, and endothelial cells [4]. Among these infiltrating immune cells, mast cells have garnered increasing attention. While traditionally recognized for their role in allergic responses, mast cells accumulate significantly in the gastric tumor stroma and correlate with poor clinical outcomes and increased microvessel density [5]. Once activated, tumor-associated mast cells can release a plethora of cytokines, proteases, and angiogenic factors, which remodel the extracellular matrix and facilitate tumor vascularization and metastasis [6]. However, the precise mechanism by which GC cells activate mast cells to adopt this pro-tumorigenic phenotype remains unknown.

Emerging evidence suggests that exosomes serve as pivotal mediators of intercellular communication within the TME [7]. By transferring bioactive cargoes, including proteins, lipids, and nucleic acids, exosomes derived from tumor cells can reprogram recipient stromal cells to foster a permissive niche for malignancy [8]. Recent studies indicate that GC-derived exosomes can modulate the behavior of macrophages and fibroblasts [9]. However, their specific role in modulating mast cell activation requires further investigation. The Stem Cell Factor (SCF)/c-KIT signaling axis is an important signaling pathway that regulates the activation and degranulation of mast cells. c-KIT (CD117) is a receptor tyrosine kinase highly expressed on mast cells, regulating their survival, proliferation, and degranulation [10]. SCF, the ligand for c-KIT, is often upregulated in various cancers and is known to drive stromal cell activation [11]. The activation of the mast cell SCF/c-KIT signaling pathway in the cancer microenvironment was proven to be conducive to cancer metastasis [12]. Therefore, we hypothesize that GC cells may utilize exosomes to deliver SCF or upstream regulators to the TME, thereby activating MCs via the SCF/c-KIT pathway to support tumor progression.

In the present study, we aim to characterize the interaction between GC cells and mast cells mediated by tumor-derived exosomes. We investigate whether GC-derived exosomes trigger the SCF/c-KIT signaling pathway in MCs and evaluate the subsequent effects on GC metastasis and angiogenesis. Our findings will provide novel insights into the exosomal communication network within the gastric TME and highlight the SCF/c-KIT axis as a potential therapeutic target for GC metastasis.

## 2 Materials and Methods

### 2.1 Cell Culture

Human embryonic kidney cells (HEK-293T) (Procell, CL-0005, Wuhan, China), human gastric epithelial cells (GES-1) (Procell, CL-0563), human gastric adenocarcinoma cells (AGS) (Procell, CL-0022), human gastric carcinoma cells (MKN1) (Procell, CL-0982), human umbilical vein endothelial cells (HUVEC) (Procell, CL-0675), and mouse gastric carcinoma cells (MFC) (Procell, CL-0156), were commercially obtained from Procell Life Science & Technology Co., Ltd. Human mast cells (LAD2) (CVCL_0387) were acquired from A. Kirshenbaum and D. Metcalfe (NIH, USA). MFC cells stably expressing firefly luciferase (MFC-Luc, AOPEISAI, ORC0130LUC, Shanghai, China) were commercially obtained from AOPEISAI Biotechnology Co., Ltd. All commercially obtained cell lines were authenticated by the supplier via STR profiling. LAD2 cells were authenticated by morphological and functional characterization as previously described [13]. All cell lines tested negative for mycoplasma contamination. All cells were cultured under standard conditions in a humidified incubator at 37°C with 5% CO_2_. Each cell line was maintained in its recommended growth medium, supplemented with 10% fetal bovine serum (FBS) (Gibco, Thermo Fisher Scientific, A5256701, USA) and 1% penicillin-streptomycin (PS) (Gibco, Thermo Fisher Scientific, 15140122), and was routinely passaged upon reaching 80–90% confluence. The specific culture media were as follows: HEK-293T, GES-1, MKN1, and MFC cells were cultured in Dulbecco’s Modified Eagle Medium (DMEM) (Gibco, Thermo Fisher Scientific, 11965092). AGS cells were maintained in F-12K Medium (Gibco, Thermo Fisher Scientific, 21127022). LAD2 cells, a mast cell line, were propagated in suspension using StemPro-34 Medium (Gibco, Thermo Fisher Scientific, 10639011) containing StemPro nutritional supplement (Gibco, Thermo Fisher Scientific, 10641-025), 2 mM L-glutamine (Gibco, Thermo Fisher Scientific, 25030081), and 100 ng/mL human stem cell factor (Gibco, Thermo Fisher Scientific, AF-300-07-10UG). HUVEC cells were cultured in Endothelial Cell Medium (ECM) (Procell, CM-0675).

To verify the uptake of exosomes by LAD2 cells, exosomes were fluorescently labeled using the BeyoExo™ Exosome Labeling and Tracking Kit (PKH67) (Beyotime, C3635S, Shanghai, China) according to the manufacturer’s instructions. Then, LAD2 cells were seeded in complete medium and incubated with PKH67-labeled exosomes (40 μg/mL) for 4 h at 37°C. Cells treated with PBS were used as the control group. After incubation, cells were washed, fixed with paraformaldehyde, and nuclei were stained with DAPI. Internalization of fluorescent exosomes was visualized by confocal microscopy (OLYMPUS, Olympus FV3000 Confocal Laser Scanning Microscope, Japan).

### 2.2 Lentiviral Transduction and Stable Line Generation

A lentivirus-based short hairpin RNA (shRNA) strategy was employed using three distinct shRNA sequences to downregulate SCF expression in AGS and MKN1 cells, and three distinct shRNA sequences to downregulate TPSAB1 expression in LAD2 cells. These shRNA sequences were cloned into the lentiviral vector pLKO.1. Both the shRNA vectors and the packaging plasmids were obtained from Addgene (Beijing Zhongyuan Co., Beijing, China).

For lentivirus production, HEK-293T cells were seeded in 6-well plates at a density of 5 × 10^5^ cells per well and cultured until reaching 60–80% confluence. Cells were co-transfected with the shRNA transfer vector (pLKO.1) (Addgene, #8453) and the third-generation packaging plasmids pMDLg/pRRE (Addgene, #12251), VSV-G (Addgene, #12259), and pRSV-Rev (Addgene, #12253) at a mass ratio of 2:3:5 (REV:VSVG:PMDL) using Lipofectamine 3000 (Invitrogen, Thermo Fisher Scientific, L3000015, USA) according to the manufacturer’s instructions. The medium was refreshed 6 h post-transfection. Viral supernatants were harvested 48 h post-transfection, filtered through a 0.45-μm filter (Beyotime, FF365-10pcs), and concentrated via ultracentrifugation (50,000× *g*, 90 min, 4°C) (Beckman Coulter, Optima XPN-100, USA).

For transduction, cells were seeded in 6-well plates (Corning, 353046, USA) at a density of 2 × 10^5^ cells per well and cultured until reaching 60–70% confluency. Cells were then transduced with lentiviral particles at a multiplicity of infection (MOI) of 10 in the presence of poly-L-lysine (Sigma-Aldrich, P8920, USA; diluted to 8 μg/mL) to enhance transduction efficiency. After 12–16 h of co-culture with the virus, the medium was replaced with fresh culture medium. After 48 h, stable transductants were selected using puromycin (2 μg/mL) (Sigma-Aldrich, P9620) for two weeks. Knockdown efficiency was validated by qRT-PCR and Western blot. All procedures were conducted at 37°C with 5% CO_2_. The shRNA sequences are shown in Table 1.

**Table 1:** The shRNA sequences used for knockdown of SCF (KITLG) in AGS and MKN1 cells and Tryptase (TPSAB1) in LAD2 cells.

Name	Sense 5′-3′	Antisense 5′-3′
sh-SCF-1	CCGGGCGAGATGGTAGTACAATTGTCTCGAGACAATTGTACTACCATCTCGCTTTTT	AATTAAAAAGCGAGATGGTAGTACAATTGTCTCGAGACAATTGTACTACCATCTCGC
sh-SCF-2	CCGGGTTCATGTTTGCTTCATAAATCTCGAGATTTATGAAGCAAACATGAACTTTTT	AATTAAAAAGTTCATGTTTGCTTCATAAATCTCGAGATTTATGAAGCAAACATGAAC
sh-SCF-3	CCGGGCTTGTATCAACACTGTTACTCTCGAGAGTAACAGTGTTGATACAAGCTTTTT	AATTAAAAAGCTTGTATCAACACTGTTACTCTCGAGAGTAACAGTGTTGATACAAGC
sh-Tryptase-1	CCGGGCGTGGGACCGGACGTCAAGGCTCGAGCCTTGACGTCCGGTCCCACGCTTTTT	AATTAAAAAGCGTGGGACCGGACGTCAAGGCTCGAGCCTTGACGTCCGGTCCCACGC
sh-Tryptase-2	CCGGGGGCGATGTGGACAATGATGACTCGAGTCATCATTGTCCACATCGCCCTTTTT	AATTAAAAAGGGCGATGTGGACAATGATGACTCGAGTCATCATTGTCCACATCGCCC
sh-Tryptase-3	CCGGGGATCATCGTGCACCCACAGTCTCGAGACTGTGGGTGCACGATGATCCTTTTT	AATTAAAAAGGATCATCGTGCACCCACAGTCTCGAGACTGTGGGTGCACGATGATCC

### 2.3 Isolation and Characterization of Exosomes

Exosomes were isolated from the conditioned medium of gastric epithelial GES-1 cells and gastric carcinoma MKN1 and AGS cells. Briefly, cells were cultured to nearly 80% confluence in media containing 10% exosome-depleted FBS (Gibco, Thermo Fisher Scientific, A2720801), followed by a 48-h incubation in fresh exosome-depleted medium to collect conditioned supernatant. Exosomes were purified by differential centrifugation. Sequential steps at 4°C at 300× *g* (for 10 min), 2000× *g* (for 10 min), and 10,000× *g* (for 30 min) were performed to remove cells, debris, and large vesicles, followed by final pelleting via ultracentrifugation at 4°C at 145,000× *g* for 70 min (Optima XPN-100, Beckman, USA). The pellet was washed and resuspended in PBS. Exosome protein concentration was quantified using a BCA protein assay kit (Biosharp, BL521A, Hefei, China). For molecular characterization, exosome markers (CD9, CD63, CD81, TSG101) were detected by Western blot, and Calnexin was the negative marker. The size distribution and concentration of purified exosomes were analyzed by nanoparticle tracking analysis (NTA) (Malvern Panalytical Ltd., NanoSight NS300 system, UK). For morphological examination, exosomes were adsorbed onto formvar-carbon-coated grids, negatively stained with 2% uranyl acetate, and visualized under a transmission electron microscope (TEM) (Hitachi High-Tech Corporation, HT7800, Japan) at 80 kV.

### 2.4 RNA Isolation and Quantitative Reverse-Transcription PCR (qRT-PCR) Assay

Total RNA extraction was performed from cultured cells with TRIzol reagent (Invitrogen, Thermo Fisher Scientific, 15596026), following the standard protocol. In brief, cells were directly lysed in the culture dish using TRIzol, and chloroform (Sigma-Aldrich, 288306) was added for phase separation. The aqueous phase containing RNA was mixed with isopropanol (Sigma-Aldrich, I9516) for precipitation. The resulting RNA pellet was washed with 75% ethanol (Sigma-Aldrich, E7023), air-dried, and finally resuspended in RNase-free water (Invitrogen, Thermo Fisher Scientific, 10977035). RNA concentration and purity were determined using a NanoDrop 2000 spectrophotometer (Thermo Fisher Scientific, USA). Only RNA samples with A260/A280 ratios between 1.8 and 2.2 and A260/A230 ratios between 2.0 and 2.2 were used for subsequent analyses. RNA integrity was assessed by agarose gel electrophoresis, and only samples with clear 28 S and 18 S ribosomal RNA bands were subjected to reverse transcription. For reverse transcription, 1 μg of total RNA from each sample was converted to cDNA using the PrimeScript RT kit (Takara, RR037A, Japan) under the following conditions: 37°C for 15 min and 85°C for 5 s. The qRT-PCR was subsequently conducted using SYBR Green master mix (Yeasen, 11201ES03, China). All qPCR reactions were performed in a total volume of 20 μL, containing 10 μL of 2× SYBR Green Master Mix, 2 μL of cDNA template, 0.4 μL each of forward and reverse primers (10 μM), and 7.2 μL of nuclease-free water. All reactions were run in triplicate on a StepOnePlus™ Real-Time PCR System (Applied Biosystems, Thermo Fisher Scientific) under the following cycling conditions: 95°C for 5 min for initial polymerase activation and hot-start enzyme release, followed by 40 cycles of 95°C for 10 s and 60°C for 30 s, with a melt-curve analysis step (95°C for 15 s, 60°C for 1 min, 95°C for 15 s) to confirm amplicon specificity. All qRT-PCR experiments were performed with three independent biological replicates (n = 3). For each biological replicate, each sample was run in triplicate technical replicates. The threshold cycle (Ct) values were automatically determined using the instrument’s software. Primer sequences for all target genes are provided in Table 2. The relative mRNA expression levels were normalized to the endogenous control GAPDH and calculated using the 2^−ΔΔCt^ method. Data are presented as mean ± SD from the three biological replicates.

**Table 2:** Primers for qRT-PCR.

Gene	Forward (5′-3′)	Reverse (5′-3′)
SCF (KITLG)	AATCCTCTCGTCAAAACTGAAGG	CCATCTCGCTTATCCAACAATGA
Tryptase (TPSAB1)	ACCACATTTGTGACGCAAAATAC	CCAGTCCAAGTAGTAGGTGACAC
VEGF (VEGFA)	AGGGCAGAATCATCACGAAGT	AGGGTCTCGATTGGATGGCA
GAPDH	GGAGCGAGATCCCTCCAAAAT	GGCTGTTGTCATACTTCTCATGG

### 2.5 Western Blot

For western blot analysis, total protein was isolated from cultured cells or tissue specimens with IP lysis buffer (Beyotime, P0013) containing protease and phosphatase inhibitor cocktails (Beyotime, P1046). Protein concentrations were quantified using a bicinchoninic acid (BCA) protein assay kit (Beyotime, P0010). Equal amounts of protein (40 μg per lane) were resolved by 10% SDS-PAGE and subsequently electro-transferred onto nitrocellulose membranes. After blocking with 5% non-fat milk for 1 h at room temperature, the membranes were probed overnight at 4°C with the following primary antibodies diluted in blocking buffer: anti-CD81 (A5270, 1:500, ABclonal, Wuhan, China), anti-CD9 (A1703, 1:2000, ABclonal), anti-TSG101 (A1692, 1:500, ABclonal), anti-CD63 (ab134045, 1:1000, Abcam, UK), anti-Calnexin (A15631, 1:1000, ABclonal), anti-SCF (sc-13126, 1:200, Santa, USA), anti-β-actin (AC038, 1:10000, ABclonal), anti-Tryptase (A19801, 1:1000, ABclonal), anti-p-ERK (80031-1-RR, 1:2000, Proteintech, USA), anti-ERK (66192-1-IG, 1:2000, Proteintech), anti-p-p38 (12874-1-AP, 1:500, Proteintech), anti-p38 (66234-1-IG, 1:2000, Proteintech), anti-p-JNK (AP1337, 1:1000, ABclonal), anti-JNK (A4867, 1:1000, ABclonal). For total protein samples from cells and tissues, β-actin was used as the loading control. For exosome samples, CD9 was used as both a positive exosome marker and a loading reference. Following three washes with PBST, membranes were incubated for 1 h at room temperature with horseradish peroxidase (HRP)-conjugated goat anti-rabbit IgG (H+L) (SA00001-2, 1:5000, Proteintech) for rabbit primary antibodies, or HRP-conjugated goat anti-mouse IgG (H+L) (SA00001-1, 1:5000, Proteintech) for mouse primary antibodies. Immunoreactive bands were detected using an enhanced chemiluminescence (ECL) substrate (Beyotime, P0018M) and captured with a Tanon 5200 gel imaging system. Band intensity was analyzed densitometrically using ImageJ software (version 1.53, National Institutes of Health, USA). The number of biological replicates for each group was n = 3. For phosphorylation analysis, the band intensity of each phosphorylated protein was first normalized to its corresponding total protein (e.g., p-ERK/ERK, p-p38/p38, p-JNK/JNK), and then the normalized values were used for statistical comparisons between groups.

### 2.6 Degranulation Assay of β-Hexosaminidase Release Rate

LAD2 mast cells were seeded in a 24-well plate at 5 × 10^5^ cells/mL (0.5 mL per well). As LAD2 cells are non-adherent and grow in suspension, plates were gently centrifuged at 200× *g* for 5 min to settle cells to the bottom, followed by a 30-min resting period at 37°C to allow cell equilibration. Cells were then stimulated with various treatments for 24 h at 37°C. PBS served as the negative control, and 50 ng/mL SCF as the positive control; all exosome treatments were applied at a concentration of 40 μg/mL. After stimulation, 50 μL of supernatant from each well was transferred to a 96-well plate and incubated with 50 μL of substrate solution containing 1 mM p-nitrophenyl-N-acetyl-β-D-glucosaminide (Calbiochem, Merck KGaA, Germany, 487052) in 0.1 M citrate buffer (pH 4.5) at 37°C for 60 min. The reaction was stopped by adding 150 μL of glycine stop buffer (200 mmol/L, pH 10.4), and the absorbance was measured at 405 nm using a microplate reader (BioTek, ELX808, USA). The remaining supernatant was removed, and cells were lysed with 200 μL of 0.5% Triton X-100 (Sigma-Aldrich, T8787) for 30 min. The lysates were centrifuged at 10,000× *g* for 30 min at 4°C, and the absorbance of the resulting supernatant was similarly measured. β-Hexosaminidase release (%) was calculated as the absorbance of the culture supernatant divided by the total absorbance. For all measurements, the absorbance values were corrected by subtracting the background absorbance of substrate-only control wells (containing substrate and stop buffer without sample). Briquilimab (10 nM, MCE, HY-P99488, USA) was used to block c-KIT and added to the cells 1 h prior to stimulation and remained present throughout the 24-h stimulation period. Human IgG1 kappa (MCE, HY-P99001) was used as the isotype control antibody of Briquilimab under the same conditions. The number of biological replicates for each group was n = 3.

### 2.7 Enzyme-Linked Immunosorbent Assay (ELISA)

The release of key inflammatory mediators from activated LAD2 mast cells was quantified by ELISA. For stimulation experiments, cells were seeded in 24-well plates at a density of 5 × 10^5^ cells/mL in complete medium and incubated overnight to allow attachment. Cells were then stimulated with various treatments for 24 h at 37°C. Following stimulation, cell culture supernatants were collected and centrifuged at 2000× *g* for 6 min at 4°C to remove cellular debris and particulates. The clarified supernatants were aliquoted and stored at −80°C until analysis.

The levels of histamine, TNF-α, MCP-1, tryptase, IL-8, and VEGF in the supernatants were determined using commercially available ELISA kits according to the manufacturer’s instructions. The specific kits used were as follows: Human TNF-α Quantikine ELISA Kit (R&D Systems, Minneapolis, DTA00D, USA), Human CCL2/MCP-1 Quantikine ELISA Kit (R&D Systems, DCP00), Human IL-8/CXCL8 Quantikine ELISA Kit (R&D Systems, D8000C), Human VEGF Quantikine ELISA Kit (R&D Systems, DVE00), Human Histamine ELISA Kit (Abcam, ab213975) and Human Tryptase ELISA Kit (Antibodies.com, A78914, UK). Briefly, 100 μL of each standard, control, or sample was added to the appropriate wells pre-coated with capture antibodies. The plates were sealed and incubated for 2 h at room temperature on a horizontal orbital shaker at 500 rpm. After incubation, wells were washed four times with 300 μL of wash buffer using an automated plate washer (BioTek, Model 405 TS). Following washing, 100 μL of the detection antibody conjugate was added to each well, and plates were incubated for 2 h at room temperature with shaking. After an additional washing step, 100 μL of substrate solution was added to each well, and plates were incubated in the dark for 30 min at room temperature. The enzymatic reaction was terminated by adding 100 μL of stop solution (2 N H_2_SO_4_). Absorbance was measured at 450 nm using a microplate reader (BioTek, ELX808). All samples and standards were assayed in duplicate. The concentrations of each analyte were calculated from the standard curve. Only standard curves with an R^2^ value greater than 0.99 were considered acceptable for quantification. The number of biological replicates for each group was n = 3.

For the detection of the murine functional homolog of human IL-8 in mouse serum samples, a commercial ELISA kit (Mouse IL-8/CXCL1/KC ELISA Kit, Biorbyt, orb1085968, UK) was used according to the manufacturer’s instructions. Mouse CXCL1 (KC) is the functional homolog of human IL-8 (CXCL8) and plays analogous roles in inflammation and angiogenesis.

### 2.8 Transwell Chemotaxis Assay for Mast Cell Recruitment

A Transwell chamber assay was performed to evaluate the chemotactic recruitment of LAD2 cells by gastric cancer cells. Firstly, the lower chambers of 24-well plates were seeded with cells representing the following experimental groups: GES-1 (2 × 10^5^ cells per well) treated with DMSO (Sigma-Aldrich, D2650), AGS (2 × 10^5^ cells per well) and MKN1 (2 × 10^5^ cells per well) treated with DMSO, and AGS (2 × 10^5^ cells per well) and MKN1 (2 × 10^5^ cells per well) pre-treated with 10 μM GW4869 (MCE, HY-19363) for 24 h. For solvent control, GES-1, AGS, and MKN1 cells were treated with an equivalent volume of DMSO (final concentration: 0.1%, v/v). Identical DMSO concentrations were maintained across all experimental groups, including GW4869-treated groups, to ensure that any observed effects were attributable to the treatment rather than the solvent. After the lower chamber cells adhered, serum-starved LAD2 mast cells were seeded into the upper chambers of Transwell inserts (5.0 μm pore size) (Corning, 3421, USA) at a density of 2 × 10^5^ cells per insert. The co-culture system was incubated for 6 h at 37°C to allow mast cell migration toward the chemoattractants. Subsequently, non-migratory cells on the upper membrane surface were removed, and the cells that migrated to the lower surface were fixed, stained with crystal violet, and counted under a light microscope (CKX53 Inverted Microscope, Evident Corporation, Japan) from five random fields per well.

### 2.9 Tube Formation Assay

Matrigel^®^ (Corning, 354277, USA) was thawed overnight on ice at 4°C and used at its original protein concentration (~10 mg/mL) without further dilution. A volume of 150 μL of the liquid Matrigel was carefully dispensed into each well of a 48-well plate using pre-cooled pipette tips to avoid premature gelation. The plate was then incubated at 37°C for 30 min in a humidified incubator to allow complete polymerisation. After solidification, human umbilical vein endothelial cells (HUVECs) were detached with trypsin-EDTA, resuspended in endothelial cell growth medium (ECGM; PromoCell, C-22010) supplemented with 5% fetal bovine serum (FBS), 10 ng/mL vascular endothelial growth factor (VEGF), 10 ng/mL basic fibroblast growth factor (bFGF), and 1% penicillin-streptomycin, and seeded onto the Matrigel-coated wells at a density of 2 × 10^4^ cells per well in a total volume of 200 μL. Immediately after seeding, the indicated treatments (or vehicle control) were added to the culture medium. Tryptase (3 μg/mL) (MCE, HY-P71377) was used as a positive control. The plates were returned to the incubator and tube formation was assessed after 24 h using a light microscope (CX40, SOPTOP, China). Five random fields per well were photographed. All experiments were performed independently three times, with three technical replicates per condition in each experiment (n = 3 biological replicates per group).

### 2.10 Extraction of Exosomes from Gastric Cancer Tissues

To obtain tumor tissues for exosome extraction, a subcutaneous gastric cancer model was established in 6-week-old female C57BL/6 mice (body weight range: 16–20 g; n = 10 per group), which were purchased from Beijing Vital River Laboratory Animal Technology Co., Ltd. (Beijing, China). Briefly, 5 × 10^6^ MFC cells suspended in 100 μL of PBS were subcutaneously injected into the right flanks of the mice. Tumors were allowed to grow for approximately 14 days until they reached a diameter of approximately 1 cm (or until the tumor volume reached approximately 500 mm^3^). Mice were then euthanized, and tumor tissues were harvested. The collected tumor and normal gastric tissues were minced into fragments (<4 mm^3^), and homogenized in cold PBS. Sequential steps at 300× *g* (for 10 min, 4°C), 2000× *g* (for 10 min, 4°C), and 10,000× *g* (for 30 min, 4°C) were performed to remove cells, debris, and large vesicles, followed by filtration (0.22 μm) and ultracentrifugation (120,000× *g*, 70 min, 4°C) to pellet exosomes. The final pellet was resuspended in PBS and characterized by NTA, TME, and Western blot for exosomal markers.

### 2.11 Establishment of a Mouse Model for Gastric Cancer Metastasis

A pulmonary metastasis model of gastric cancer was established to assess the role of exosomes in tumor dissemination. The 6-week-old female C57BL/6 mice (body weight range: 16–20 g; n = 5 per group) were purchased from Beijing Vital River Laboratory Animal Technology Co., Ltd. (Beijing, China), and were housed under specific pathogen-free conditions at 22 ± 2°C with a 12-h light/dark cycle (lights on at 08:00). Both food and water were freely accessible. Mice were acclimatized to the animal facility for 7 days before the start of the experiment.

Briefly, 5 × 10^5^ MFC-Luc cells suspended in 100 μL of PBS were intravenously injected into the tail vein of 6-week-old female C57BL/6 mice. This route enables the cells to circulate and arrest in the lung capillaries, forming metastatic nodules. Beginning on day 10 post-inoculation, mice were randomly assigned to five intervention groups (n = 5 per group; the experimental unit was the individual mouse) using a computer-generated random number sequence (Excel, Microsoft Corporation, USA). The sample size (n = 5 per group) was determined based on a power analysis using effect sizes estimated from pilot experiments (α = 0.05, power = 0.80). All mice were included in the analysis and no animals were excluded from the study.

The groups were as follows: (1) PBS, (2) Control-Exo (exosomes derived from normal gastric tissue, 200 μg per animal), (3) MFC-Exo (exosomes derived from MFC tumors, 200 μg per animal), (4) MFC-Exo (200 μg per animal) + IgG mAb (500 μg per animal, Sigma, USA), and (5) MFC-Exo (200 μg per animal) + ACK2 (c-KIT blocking antibody, 500 μg per animal, BioXcell, USA). All treatments were administered via tail vein injection every three days for a total of five administrations. The investigator performing the injections was not blinded to group allocation; however, all outcome assessments were conducted by investigators blinded to treatment groups.

To longitudinally monitor metastatic burden, *in vivo* bioluminescence imaging was performed using an *in vivo* imaging system (IVIS) Spectrum system. Mice were anesthetized with isoflurane (2–3% in oxygen) during each imaging session. For bioluminescence imaging, mice were intraperitoneally injected with D-luciferin (150 mg/kg, PerkinElmer, 122799, USA) 10 min prior to imaging. The initial imaging session was conducted on the first day of treatment (day 10). Subsequent imaging sessions were carried out at five-day intervals (days 15, 20, 25, 30, and 35), for a total of six time points. Bioluminescence signals were quantified as total photon flux (photons/s) using Living Image software (PerkinElmer, version 4.7.4). The primary outcome measure was the number of surface metastatic foci on the lungs. Secondary outcome measures included *in vivo* bioluminescence imaging signal intensity and histological confirmation of metastasis.

At the experimental endpoint (day 35), all mice were euthanized by CO_2_ asphyxiation followed by cervical dislocation. Lungs were harvested, and the surface metastatic foci were counted macroscopically. The lung tissues were then either fixed for histological analysis to confirm metastasis by hematoxylin and eosin (H&E) staining. Statistical analyses were performed using GraphPad Prism software (Version 9.0, GraphPad Software, Inc., California, USA; now part of Dotmatics). Data are presented as mean ± SD. Comparisons among multiple groups were analyzed by one-way ANOVA followed by Tukey’s *post hoc* test. A *p*-value < 0.05 was considered statistically significant.

The animal study protocol was approved by the Laboratory Animal Welfare and Ethics Committee of the First Hospital of Shanxi Medical University, under approval number NO. DWYJ-2023-065. All *in vivo* assessments were performed by independent investigators who were blinded to the experimental group assignments.

### 2.12 Histological Analysis

Formalin-fixed tissue samples were embedded in paraffin and sectioned at a thickness of 4 μm for histological evaluation. All stained sections were examined and imaged under a light microscope (CX40, SOPTOP, Sunny Instruments Co., Ltd., Ningbo, China). Paraffin-embedded tissue blocks were sectioned using a rotary microtome (Leica RM2235, Leica Microsystems, Germany). Sections were floated in a 40°C water bath, mounted onto poly-L-lysine-coated glass slides (BosterBio, AR1065, USA), and dried overnight at 37°C. For staining, sections were deparaffinized in xylene and rehydrated through a graded ethanol series followed by distilled water rinse. Antigen retrieval was performed where indicated by heating sections in 10 mM sodium citrate buffer (pH 6.0) (Biologix Group, SCB-500, USA) or Tris-EDTA buffer (pH 9.0) (Scytek, TES500, USA) in a pressure cooker for 3 min at full pressure (121°C), followed by slow cooling to room temperature.

For routine histological evaluation, rehydrated sections were stained using a standard H&E staining kit (Servicebio, G1005, China) according to the manufacturer’s protocol. Briefly, sections were stained with Harris hematoxylin for 5 min, rinsed in running tap water for 5 min, differentiated in 1% acid alcohol for 2–3 s, blued in 0.2% ammonia water for 30 s, and counterstained with 1% eosin Y solution for 2 min. After dehydration through graded ethanol and clearing in xylene, sections were coverslipped with neutral mounting medium (Beyotime, C0173–100 mL). For histological quantification, five random fields per section at 200× magnification were analyzed.

For immunohistochemistry (IHC) staining of c-KIT (YR145, rabbit monoclonal, 1:400, Abcam) and Tryptase (19523, rabbit monoclonal, 1:400, Cell Signaling Technology, USA), rehydrated sections underwent antigen retrieval as described above. Endogenous peroxidase activity was blocked by incubating sections in 3% hydrogen peroxide in methanol for 10 min at room temperature in the dark. Sections were then incubated with 5% normal goat serum in PBS containing 0.1% Tween-20 (PBST) for 1 h at room temperature to block non-specific binding. After blocking, sections were incubated overnight at 4°C with the respective primary antibodies diluted in antibody diluent (Dako, S3022, USA). Following three washes with PBST (5 min each), sections were incubated with a horseradish peroxidase (HRP)-conjugated goat anti-rabbit secondary antibody (P0448, 1:200, Dako) for 1 h at room temperature. The signal was developed using a DAB Peroxidase Substrate Kit (Maxin, DAB-0031, China) with incubation times optimized for each antibody (2–5 min, monitored under microscope). Nuclei were lightly counterstained with Mayer’s hematoxylin (Dako, S3309) for 30 s. c-KIT and Tryptase were detected on separate sections to evaluate their expression levels across different treatment groups. The staining was assessed qualitatively by evaluating staining intensity (graded as negative, weak, moderate, or strong) and the proportion of positively stained cells. Group comparisons were based on qualitative assessment performed independently by two blinded investigators. No formal H-score or other quantitative scoring was applied, as the differences between groups were sufficiently pronounced to be reliably distinguished by qualitative evaluation. All assessments were performed by two independent investigators blinded to group assignments.

For immunofluorescence (IF) co-staining of CD31 (AF3628, goat polyclonal, 1:200, R&D Systems, USA) and p-ERK (4377, rabbit monoclonal, 1:200, Cell Signaling Technology, USA), sections were processed through deparaffinization, rehydration, and antigen retrieval as described above. After blocking with 5% normal donkey serum in PBST for 1 h at room temperature, sections were incubated with a mixture of the two primary antibodies (diluted in blocking buffer) overnight at 4°C in a humidified chamber. The following day, sections were washed three times with PBST (5 min each) and incubated with a mixture of secondary antibodies: Alexa Fluor 594-conjugated donkey anti-goat IgG (H+L) (705-585-147, 1:200, Jackson ImmunoResearch, USA) and Alexa Fluor 488-conjugated goat anti-rabbit IgG (H+L) (ab150077, 1:500, Abcam), for 45 min at room temperature in the dark. Cell nuclei were counterstained with DAPI (1 μg/mL, Beyotime, C1005) for 5 min. After a final wash, sections were mounted with anti-fade mounting medium (Beyotime, P0126) and sealed with nail polish. Fluorescence images were captured using a fluorescence microscope (IX35, Olympus, Japan).

### 2.13 Statistical Analysis

Quantitative results are expressed as mean ± standard deviation (SD), derived from a minimum of three biologically independent replicates. GraphPad Prism software (Version 9.0, GraphPad Software, Inc., California, USA; now part of Dotmatics) was utilized for all statistical analyses and graphical presentations. An unpaired, two-tailed Student’s *t*-test was applied to assess differences between two independent groups, provided that the data satisfied assumptions of normal distribution and equal variance. For comparisons involving three or more groups, one-way analysis of variance (ANOVA) was performed, with Tukey’s *post hoc* test conducted for specific group comparisons when the overall ANOVA result was significant. A *p*-value of less than 0.05 was considered statistically significant for all tests. For the *in vitro* experiments, the biological replicates are n = 3 in every group, while for the *in vivo* experiments, the biological replicates are n = 5 in every group.

To clearly indicate pairwise comparisons between multiple experimental groups in bar graphs while keeping the figure panels uncluttered, we adopted a consistent set of symbols. For pairwise comparisons between specific groups in the bar graphs, the statistical symbols are defined relative to the control bars: asterisks (*) denote comparisons with the first bar, number signs (#) with the second bar, triangles (▲) with the third bar, and downward triangles (▼) with the fourth bar. The same symbol definitions apply uniformly across all figures. A value of *p* < 0.05 was considered to indicate statistical significance.

## 3 Results

### 3.1 Characterization of Exosomes

**Figure 1:**
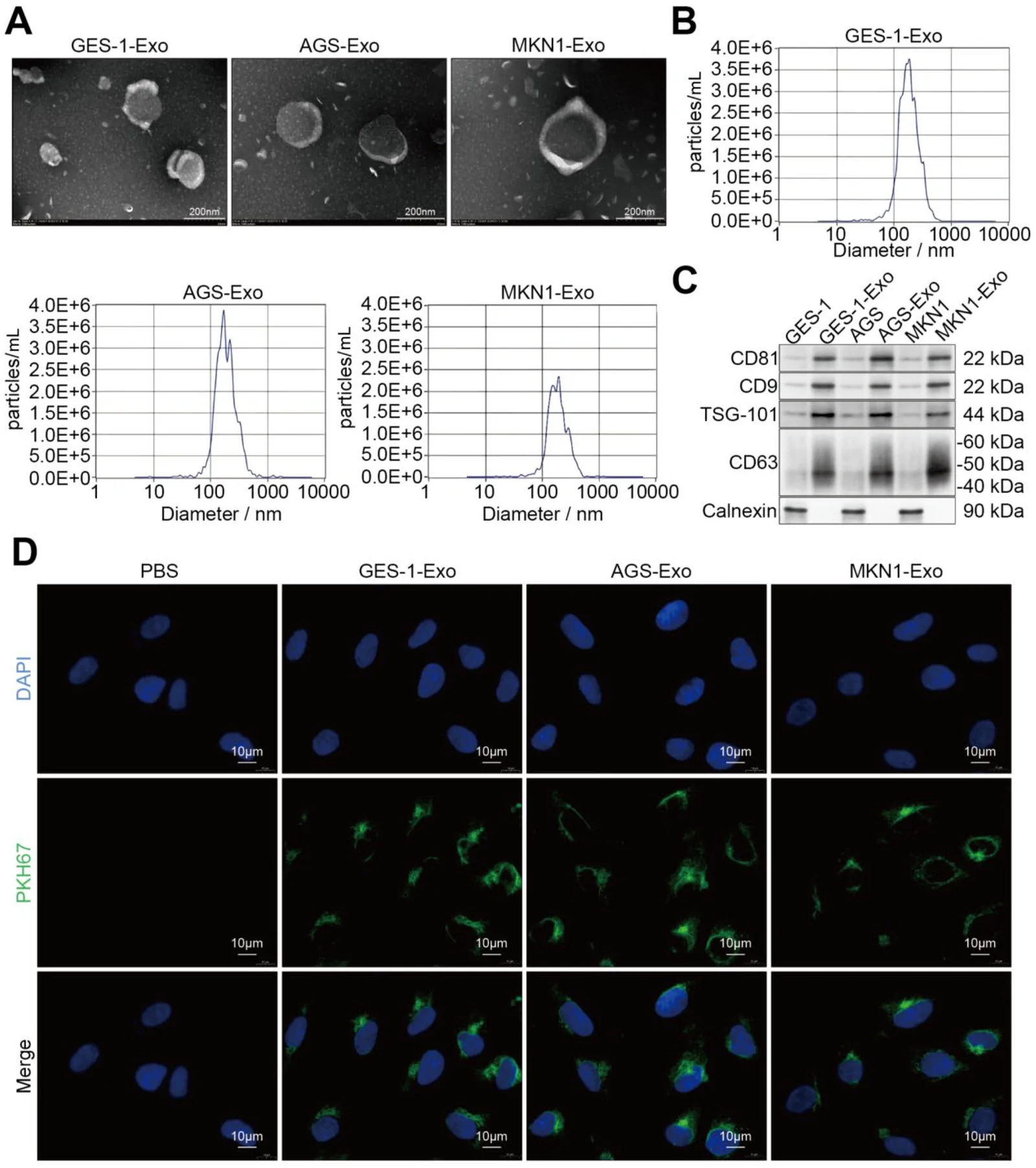
**Characterization of exosomes.** (**A**) The electron micrograph image of the exosomes derived from the supernatant of GES-1, AGS, and MKN1 cells. (GES-1-Exo, AGS-Exo, MKN1-Exo) (Bar = 200 nm). (**B**) The sizes of GES-1-Exo, AGS-Exo, and MKN1-Exo were analyzed using NTA. (**C**) Western blot analysis of GES-1-Exo, AGS-Exo, and MKN1-Exo. CD81, CD9, TSG101, and CD63 were exosomal markers. Calnexin was the negative marker. Cells were used as a control. (**D**) Fluorescence microscopy image showing the uptake of PKH67-labeled GES-1-Exo, AGS-Exo, and MKN1-Exo in LAD2 mast cells (Bar = 10 μm). Abbreviations: exosome (Exo).

Isolated exosomes from the conditioned medium of gastric epithelial cell GES-1 and GC cells AGS and MKN1 exhibited a characteristic cup-shaped morphology under transmission electron microscopy (Fig. 1A). The NTA results revealed that the majority of the vesicles had diameters ranging from 40 to 200 nm. (Fig. 1B). Western blot analysis confirmed the positive expression of exosomal markers (CD81, CD9, TSG101, and CD63) in the exosome fractions, while the endoplasmic reticulum protein calnexin was detected only in the whole-cell lysates but not in the exosomes (Fig. 1C). Furthermore, IF demonstrated that the isolated exosomes were effectively internalized by LAD2 mast cells (Fig. 1D). These results collectively confirm the successful isolation and typical characteristics of the exosomes used in this study.

### 3.2 Gastric Cancer Cells Recruited Mast Cells and Promoted Mast Cell Degranulation by Releasing SCF through Exosomes

**Figure 2:**
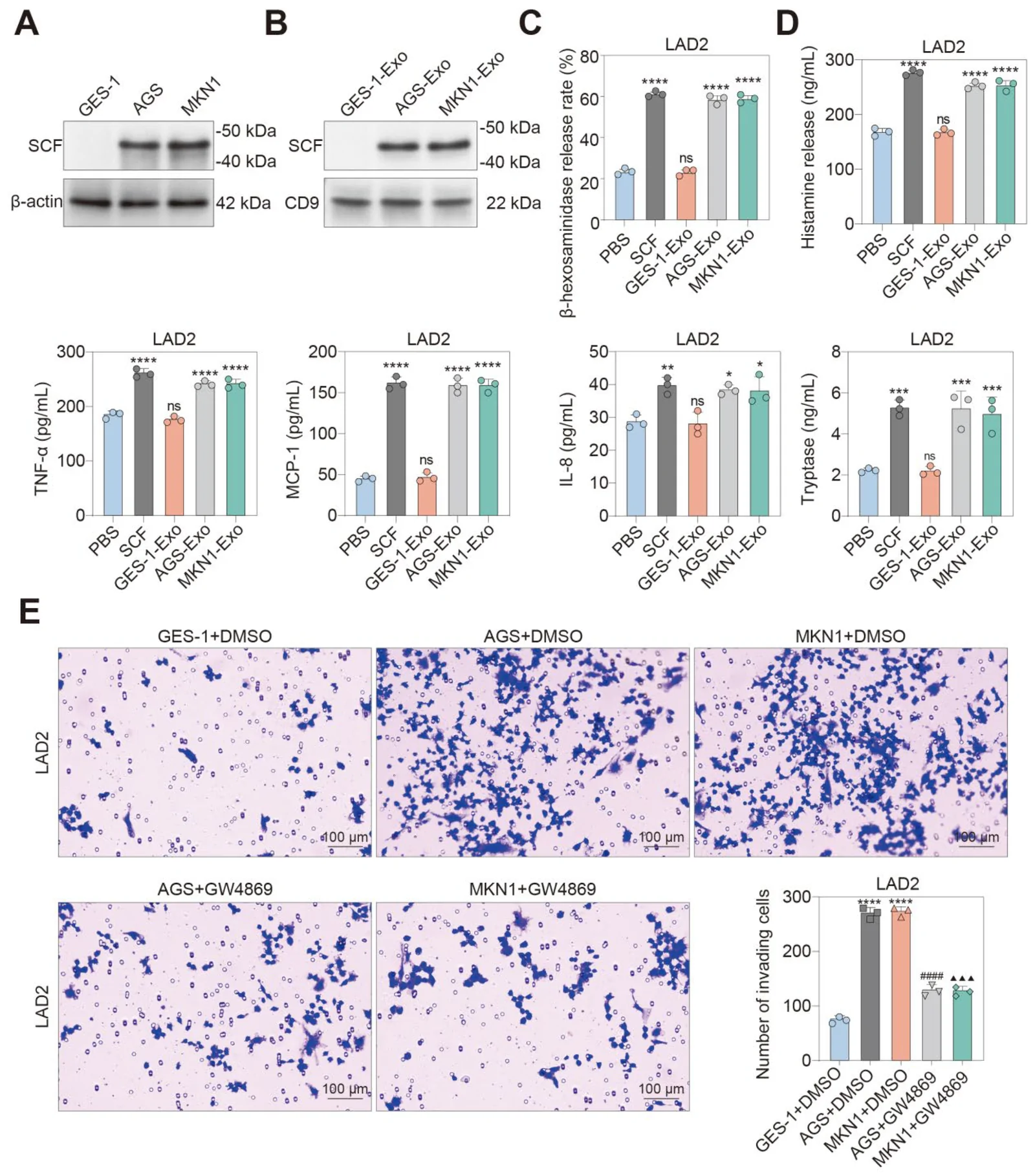
**Gastric cancer cells recruited mast cells and promoted mast cell degranulation by releasing SCF through exosomes.** (**A**) Western blot analysis of SCF and β-actin in GES-1, AGS, and MKN1 cells. (**B**) Western blot analysis of SCF and CD9 in GES-1-Exo, AGS-Exo, and MKN1-Exo. (**C**) Detection of β-hexosaminidase release in LAD2 mast cells after treatment with PBS, SCF (50 ng/mL), GES-1-Exo (40 μg/mL), 40 μg AGS-Exo (40 μg/mL), 40 μg MKN1-Exo (40 μg/mL). (**D**) Concentration of histamine, TNF-α, MCP-1, Tryptase, and IL-8 in mast cell supernatant was assessed by ELISA after different treatments. (**E**) The recruitment of LAD2 mast cells after co-culture with GES-1 + DMSO, AGS + DMSO, MKN1 + DMSO, AGS + GW4869 (10 μM), MKN1 + GW4869 (10 μM) (Bar = 100 μm). The ns indicated no significance, *p < 0.05, **p < 0.01, ***p < 0.001, ****p < 0.0001, ▲▲▲*p* < 0.001, ####*p* < 0.0001. Abbreviations: exosome (Exo).

Western blot analysis revealed that SCF was highly expressed in GC cell lines AGS and MKN1 compared to the gastric epithelial cell line GES-1 (Fig. 2A). Importantly, this high level of SCF was also detected in exosomes isolated from the conditioned medium of AGS and MKN1 cells (AGS-Exo, MKN1-Exo), while it was not in exosomes from GES-1 cells (GES-1-Exo) (Fig. 2B). To determine whether exosomes from GC cells influence mast cells, AGS-Exo and MKN1-Exo were used to treat LAD2 mast cells. The results showed that AGS-Exo and MKN1-Exo significantly induced mast cell degranulation, as measured by the release of β-hexosaminidase in cell supernatants, whereas GES-1-Exo had a minimal effect (Fig. 2C). 50 ng/mL SCF was used as a positive control. Consistent with this, ELISA assays showed that treatment with AGS-Exo or MKN1-Exo promoted production of histamine, TNF-α, MCP-1, tryptase, and IL-8 in mast cell supernatants (Fig. 2D). Furthermore, Transwell migration assays demonstrated that both AGS and MKN1 cells potently recruited mast cells. This recruitment was significantly attenuated when the tumor cells were pre-treated with the exosome secretion inhibitor GW4869, indicating a critical role for exosomes in this process (Fig. 2E).

### 3.3 Gastric Cancer Cell Exosomes Promoted Mast Cell Recruitment and Degranulation by Activating the SCF/c-KIT Signal Pathway

**Figure 3:**
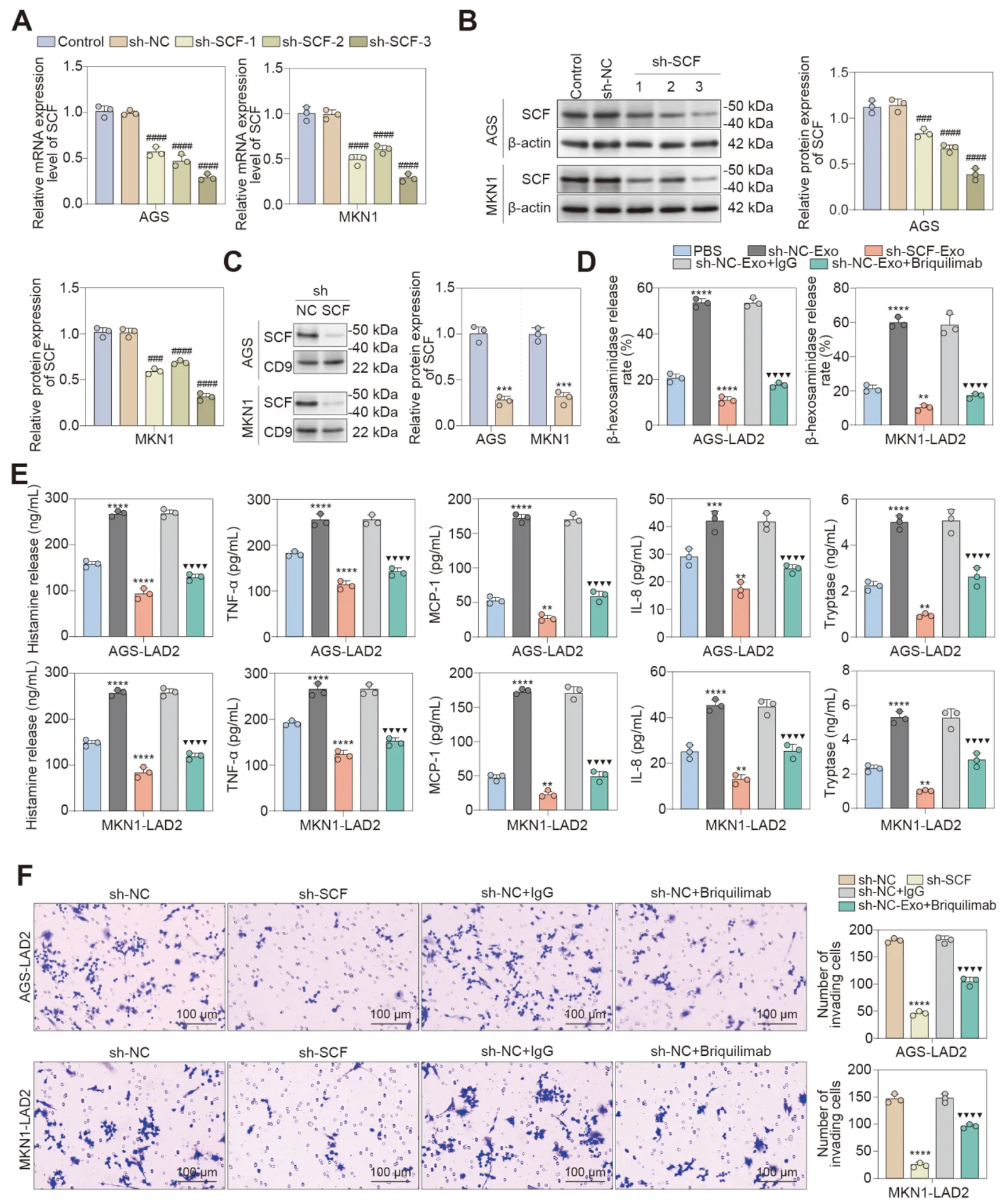
**Gastric cancer cell exosomes promoted mast cell recruitment and degranulation by activating the SCF/c-KIT signal pathway.** (**A**) qRT-PCR analysis of SCF expression in AGS and MKN1 cells transfected with sh-SCF or sh-NC. (**B**) Western blot analysis of SCF expression in AGS and MKN1 cells after transfection with sh-SCF or sh-NC. (**C**) Western blot analysis of SCF expression in exosomes derived from AGS and MKN1 cells after transfection with sh-SCF or sh-NC (sh-SCF-Exo, sh-NC-Exo). (**D**) Detection of β-hexosaminidase release in LAD2 mast cells after different treatments. (AGS and MKN1: PBS, sh-NC-Exo (40 μg/mL), sh-SCF-Exo (40 μg/mL), sh-NC-Exo (40 μg/mL) + IgG, sh-NC-Exo (40 μg/mL) + Briquilimab (10 nM)). (**E**) Concentration of histamine, TNF-α, MCP-1, Tryptase, and IL-8 in mast cell supernatant was assessed by ELISA after different treatments. (**F**) The recruitment of LAD2 mast cells after co-culture with AGS or MKN1 in different groups (sh-NC, sh-SCF, sh-NC + IgG, sh-NC + Briquilimab (10 nM)) (Bar = 100 μm). **p < 0.01, ***p < 0.001, ****p < 0.0001, ###*p* < 0.001, ####*p* < 0.0001, ▼▼▼▼*p* < 0.0001. Abbreviations: exosome (Exo).

To further investigate the functional role of exosomal SCF, we genetically knocked down SCF expression in AGS and MKN1 cells using lentiviral shRNA. Both qPCR and Western blot analyses confirmed the efficient knockdown of SCF at the mRNA and protein levels in the cells (Fig. 3A,B). Consequently, SCF protein levels were substantially diminished in the exosomes derived from these SCF-knockdown cells (sh-SCF-Exo) compared to those from control cells (sh-NC-Exo) (Fig. 3C). Functional assays revealed that exosomes from control cancer cells (sh-NC-Exo) potently induced LAD2 cell degranulation, as measured by β-hexosaminidase release. This effect was significantly attenuated when mast cells were treated with exosomes from SCF-knockdown cells (sh-SCF-Exo). Importantly, the degranulation triggered by sh-NC-Exo was markedly inhibited by pre-treating mast cells with a c-KIT blocking antibody, but not with an isotype control antibody (Fig. 3D). A similar pattern was observed in the secretion profile of mast cell mediators. ELISA showed that sh-NC-Exo, but not sh-SCF-Exo, promoted the release of histamine, TNF-α, MCP-1, tryptase, and IL-8. Again, this cytokine release was effectively blocked by the c-KIT blocking antibody Briquilimab (Fig. 3E). Finally, Transwell migration assays demonstrated that SCF knockdown in AGS and MKN1 cells significantly impaired their ability to recruit mast cells compared to control cells. Furthermore, the robust mast cell recruitment induced by control cancer cells was almost completely abolished by the addition of Briquilimab (Fig. 3F). Collectively, these results demonstrate that GC cell-derived exosomes promote mast cell recruitment and degranulation specifically through the activation of the SCF/c-KIT signaling pathway.

### 3.4 Gastric Cancer Cell Exosomes Induce Mast Cells to Release Tryptase, Thereby Promoting Angiogenesis via the MAPK Pathway

**Figure 4:**
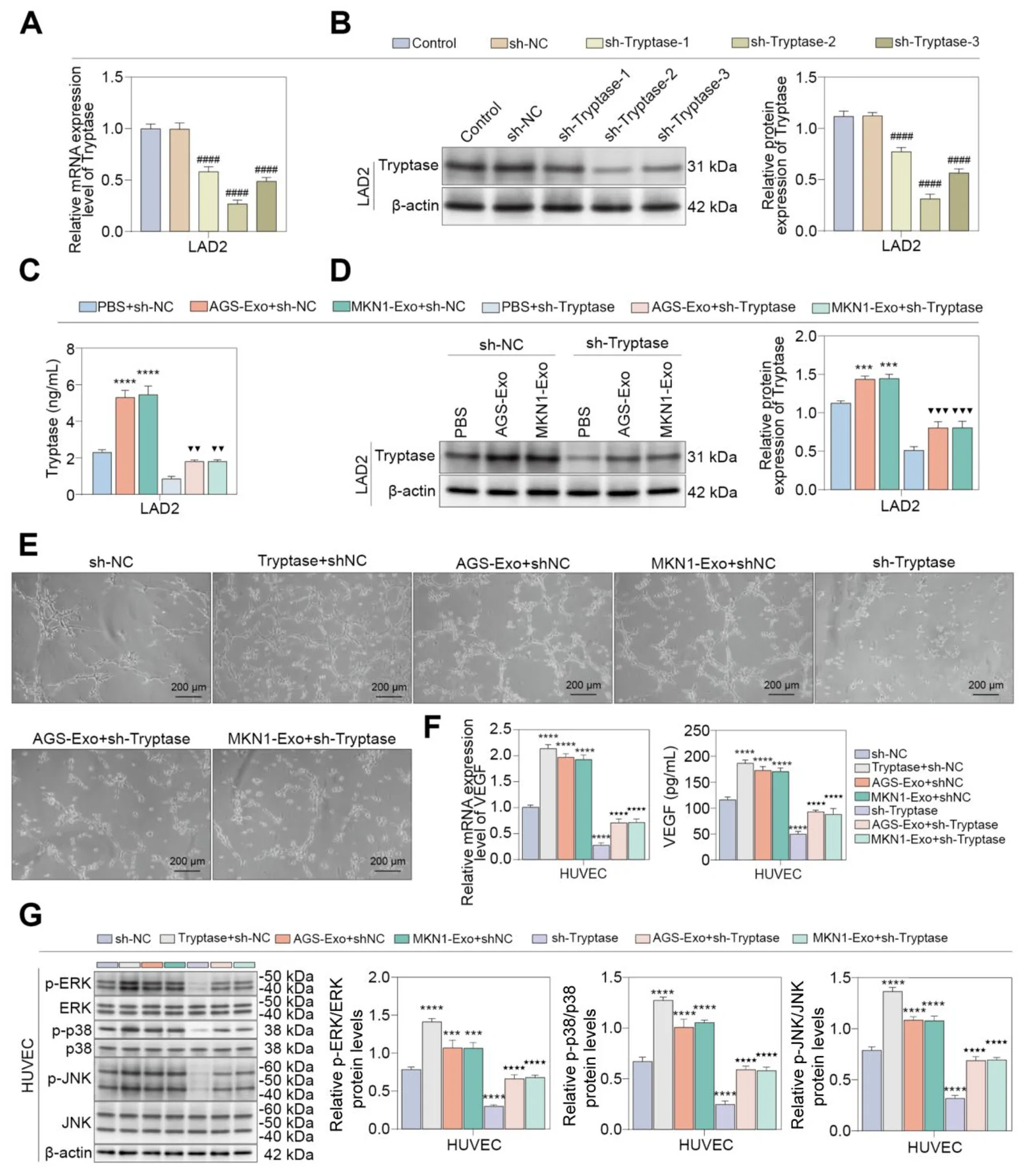
**Gastric cancer cell exosomes induce mast cells to release Tryptase, thereby promoting angiogenesis via the MAPK pathway.** (**A**) qRT-PCR analysis of Tryptase expression in LAD2 cells transfected with shRNA targeting Tryptase (sh-Tryptase) or shNC. (**B**) Western blot analysis of Tryptase expression in LAD2 cells after transfection with sh-Tryptase or shNC. (**C**) Concentration of Tryptase in mast cell supernatant assessed by ELISA in different groups. (PBS + shNC, AGS-Exo (40 μg/mL) + shNC, MKN1-Exo (40 μg/mL) + shNC, PBS + sh-Tryptase, AGS-Exo (40 μg/mL) + sh-Tryptase, MKN1-Exo (40 μg/mL) + sh-Tryptase). (**D**) Western blot analysis of Tryptase expression in LAD2 cells after different treatments. (PBS + shNC, AGS-Exo (40 μg/mL) + shNC, MKN1-Exo (40 μg/mL) + shNC, PBS + sh-Tryptase, AGS-Exo (40 μg/mL) + sh-Tryptase, MKN1-Exo (40 μg/mL) + sh-Tryptase). (**E**) Tube formation assay of HUVEC cells after treatment of mast cell supernatant in different groups (shNC, Tryptase (3 μg/mL) + shNC, AGS-Exo (40 μg/mL) + shNC, MKN1-Exo (40 μg/mL) + shNC, sh-Tryptase, AGS-Exo (40 μg/mL) + sh-Tryptase, MKN1-Exo (40 μg/mL) + sh-Tryptase). Tryptase (3 μg/mL) was used as a positive control (Bar = 200 μm). (**F**) VEGF concentration in supernatant was assessed by ELISA, and the VEGF expression level of HUVEC cells was assessed by qRT-PCR after treatment with mast cell supernatant in different groups. (**G**) Western blot analysis of p-ERK, p-p38, p-JNK, ERK, p38, JNK, and β-actin expression in HUVEC cells after treatment with mast cell supernatant in different groups. Quantitative data for phosphorylated proteins are presented as the ratio of phosphorylated protein to total protein. ***p < 0.001, ****p < 0.0001, ####*p* < 0.0001, ▼▼*p* < 0.01, ▼▼▼*p* < 0.001. Abbreviations: exosome (Exo).

To determine the specific role of mast cell-derived tryptase in GC exosome-induced angiogenesis, we first established stable tryptase-knockdown LAD2 mast cell lines using lentiviral shRNA, with knockdown efficiency confirmed at both mRNA and protein levels (Fig. 4A,B). The results of ELISA showed that treatment with AGS-Exo and MKN1-Exo significantly promoted control mast cells (sh-NC) releasing higher levels of tryptase, but tryptase-knockdown cells (sh-Tryptase) showed minimal release (Fig. 4C). Western blot analysis of cell lysates corroborated these findings (Fig. 4D). We then investigated the pro-angiogenic effect of the conditioned medium from these exosome-stimulated mast cells. Tube formation assays using HUVECs showed that conditioned medium from exosome-treated control mast cells markedly enhanced endothelial tube formation. This effect was mimicked by direct tryptase treatment and was significantly abolished when using conditioned medium from tryptase-knockdown mast cells (Fig. 4E). Furthermore, both qPCR and ELISA analyses revealed that conditioned medium from exosome-stimulated control mast cells, but not from tryptase-knockdown mast cells, potently upregulated VEGF expression in HUVECs (Fig. 4F). Mechanistically, Western blot analysis demonstrated that CM from exosome-activated control mast cells strongly induced the phosphorylation of key MAPK family members, including ERK, p38, and JNK, in HUVECs, as reflected by increased ratios of phosphorylated to total protein. This activation pattern was identical to that induced by direct tryptase treatment and was again absent when using conditioned medium from tryptase-deficient mast cells (Fig. 4G). In summary, these results indicate that tryptase released from mast cells upon GC exosome stimulation is a critical mediator that promotes angiogenesis by upregulating endothelial VEGF expression through activation of the MAPK signaling pathway.

### 3.5 Exosomes Derived from Gastric Cancer Tissues Promoted Metastasis of the Tumor through the SCF/c-KIT Signaling Pathway In Vivo

**Figure 5:**
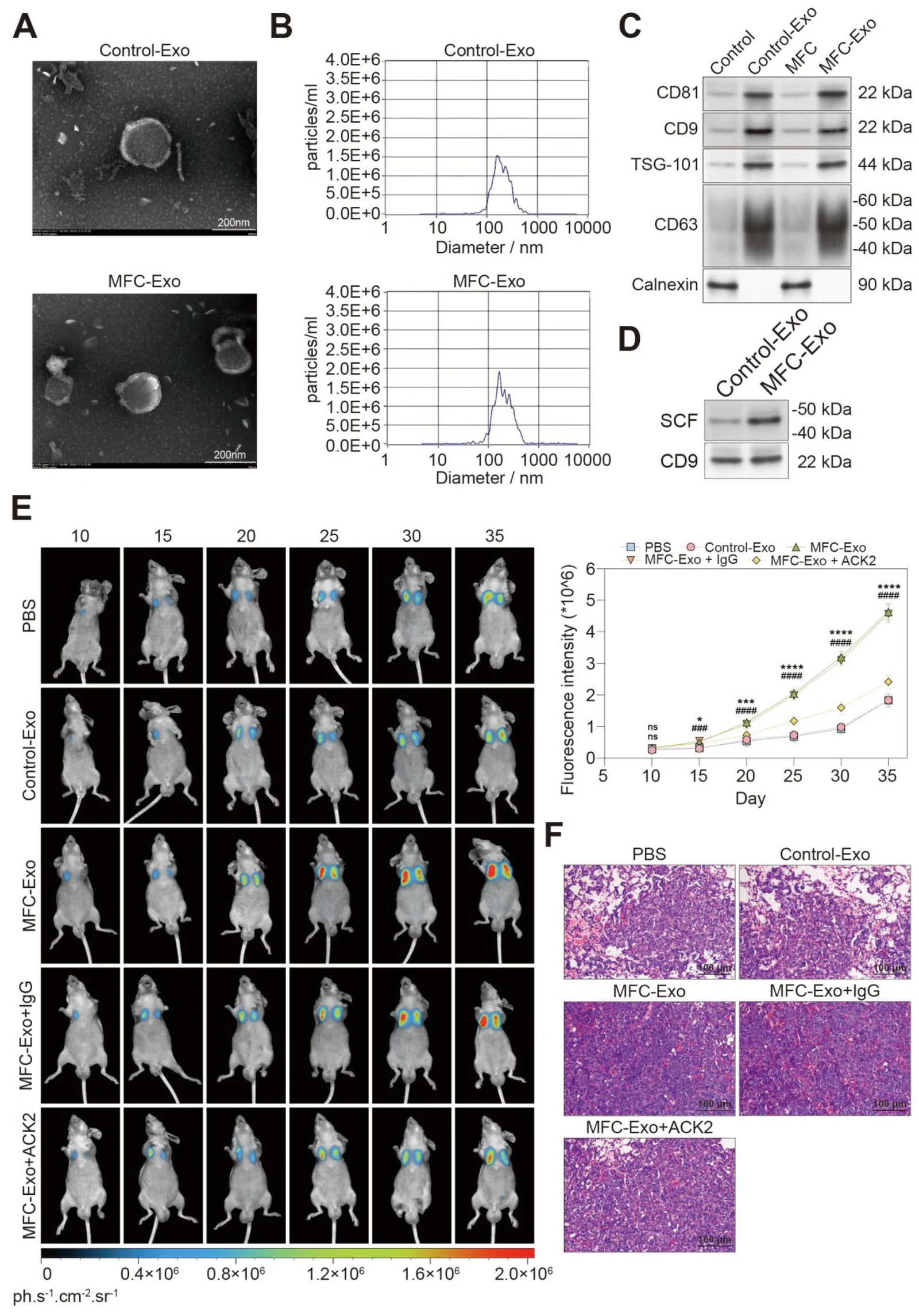
**Exosomes derived from gastric cancer tissues promoted metastasis of the tumor through the SCF/c-KIT signaling pathway *in vivo*.** (**A**) The electron micrograph image of the exosomes from gastric cancer tissue and control gastric tissue (MFC-Exo, Control-Exo) (Bar = 200 nm). (**B**) The sizes of MFC-Exo and Control-Exo were analyzed using NTA. (**C**) Western blot analysis of MFC-Exo, Control-Exo. CD81, CD9, TSG101, and CD63 were exosomal markers. Calnexin was the negative marker. Cells were used as a control. (**D**) Western blot analysis of SCF and CD9 in Control-Exo and MFC-Exo. (**E**) A gastric cancer metastasis model was established by injecting MFC cells into the tail vein of C57 mice, and exosomes or antibodies were administered via the tail vein after 10 days. The metastasis of MFC was monitored using an IVIS at 10 days, 15 days, 20 days, 25 days, 30 days, and 35 days. (**F**) Hematoxylin and eosin (H&E) staining of lung metastatic tumor 35 days after MFC cell injection (Bar = 100 μm). The ns indicated no significance, *p < 0.05, ***p < 0.001, ****p < 0.0001, ###*p* < 0.001, ####*p* < 0.0001. Abbreviations: exosome (Exo).

Next, we validated the effect of GC exosomes *in vivo*. Exosomes from both normal gastric tissues (Control-Exo) and gastric tumor tissues (MFC-Exo) were isolated from mouse models. The isolated vesicles exhibited the typical cup-shaped morphology under TEM (Fig. 5A) and had a size distribution characteristic of exosomes as measured by NTA (Fig. 5B). Western blot analysis confirmed the enrichment of exosomal markers (CD81, CD9, TSG101, CD63) and the absence of the negative marker calnexin in the exosome fractions, while calnexin was present in the parent tissue lysates (Fig. 5C). Critically, the SCF protein was abundantly present in MFC-Exo but rare in Control-Exo (Fig. 5D). We then established a lung metastasis model by intravenous injection of MFC cells. Treatment with MFC-Exo significantly accelerated the progression of lung metastases compared to the PBS or Control-Exo groups, as monitored by longitudinal bioluminescence imaging from day 10 to 35 post-injection. This pro-metastatic effect was not affected by a control antibody but was substantially attenuated when mice were co-treated with a c-KIT blocking antibody (Fig. 5E). Histological examination of lung tissues at the endpoint showed a markedly higher metastatic burden in the MFC-Exo group, which was also specifically inhibited by the c-KIT blocking antibody (Fig. 5F). These *in vivo* results demonstrate that exosomes derived from GC tissues, which carry SCF, robustly promote tumor metastasis, and this effect is dependent on the SCF/c-KIT signaling axis.

### 3.6 Exosomes Derived from Gastric Cancer Tissues Activated Mast Cell Degranulation through the SCF/c-KIT Signaling Pathway, Thereby Promoting Angiogenesis In Vivo

**Figure 6:**
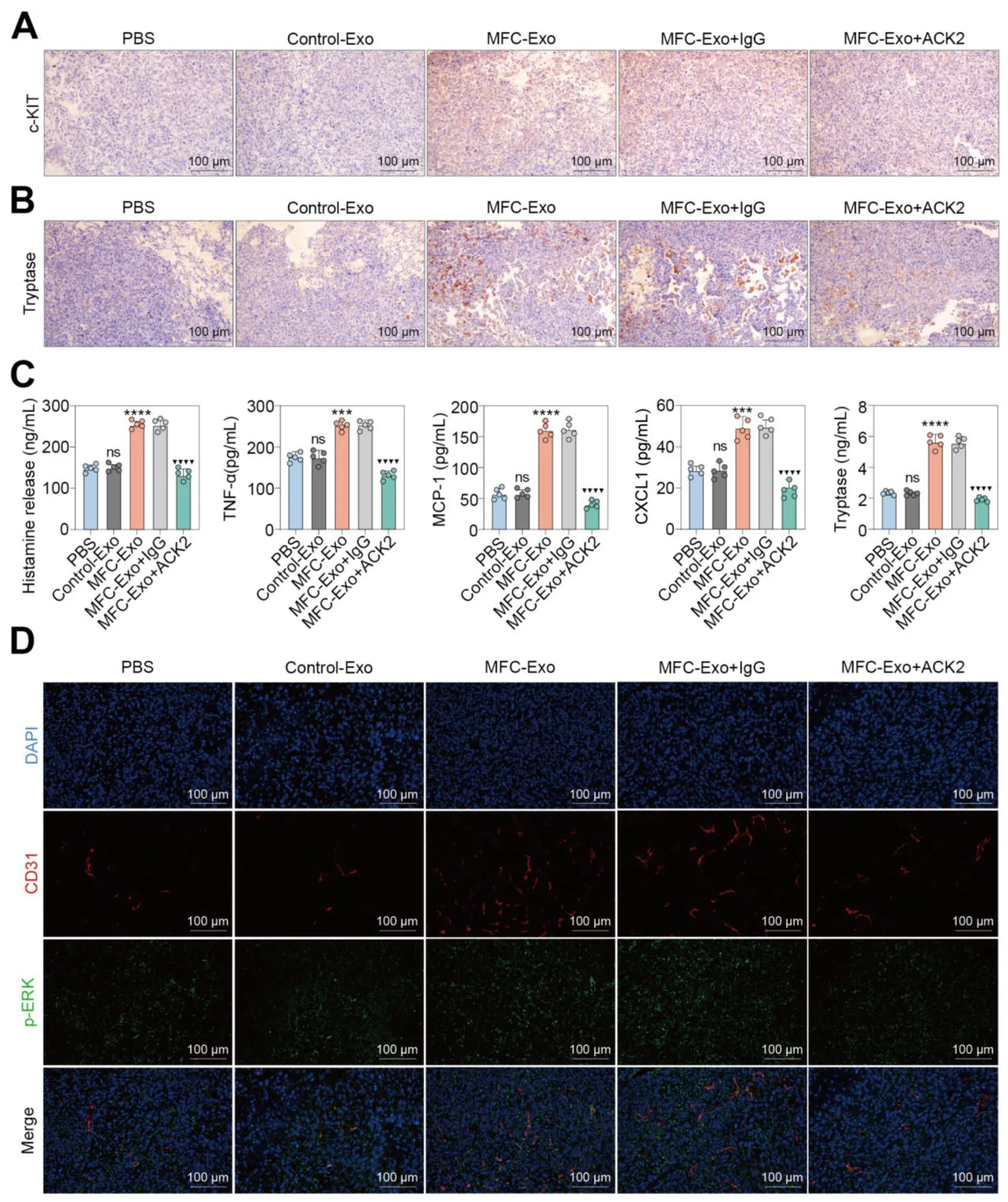
**Exosomes derived from gastric cancer tissues activated mast cell degranulation through the SCF/c-KIT signaling pathway, thereby promoting angiogenesis *in vivo*.** (**A**) Immunohistochemistry (IHC) staining of c-KIT in lung metastatic tumor 35 days after MFC cell injection (Bar = 100 μm). (**B**) IHC staining of Tryptase in lung metastatic tumor 35 days after MFC cell injection (Bar = 100 μm). (**C**) Concentration of histamine, TNF-α, MCP-1, Tryptase, and CXCL1 in mouse serum assessed by ELISA. (**D**) immunofluorescence (IF) staining of CD31 and p-ERK in lung metastatic tumor 35 days after MFC cell injection (Bar = 100 μm). The ns indicated no significance, ***p < 0.001, ****p < 0.0001, ▼▼▼▼*p* < 0.0001.

We next investigated the functional impact of tissue-derived exosomes on mast cells and angiogenesis *in vivo*. Immunohistochemical analysis of lung metastatic lesions revealed a significant increase in c-KIT-positive mast cell infiltration in mice treated with MFC-Exo compared to those receiving PBS or Control-Exo. This enhanced recruitment was specifically inhibited by co-administration of a c-KIT blocking antibody (Fig. 6A). Consistent with mast cell activation, tryptase staining was markedly elevated in the MFC-Exo group, and this effect was also abolished by c-KIT blockade (Fig. 6B). Systemic evidence of degranulation was confirmed by ELISA, which showed that serum levels of mast cell-derived mediators, including histamine, TNF-α, MCP-1, tryptase, and CXCL1, were significantly higher in the MFC-Exo group. The release of these factors was effectively suppressed by the c-KIT blocking antibody (Fig. 6C). Finally, to assess the pro-angiogenic outcome, we evaluated microvessel density in the metastatic foci by co-staining for CD31 and phosphorylated ERK. Treatment with MFC-Exo resulted in a pronounced increase in both CD31-positive vessels and p-ERK signal within the vascular endothelium. This pro-angiogenic effect was not observed in the control groups and was again significantly mitigated by the c-KIT blocking antibody (Fig. 6D). Collectively, these *in vivo* findings demonstrate that GC tissue-derived exosomes orchestrate a tumor-promoting microenvironment by recruiting and activating mast cells via the SCF/c-KIT pathway, leading to the release of pro-angiogenic factors and subsequent enhancement of intratumoral angiogenesis.

## 4 Discussion

In this study, we delineated a novel intercellular communication pathway within the GC microenvironment, where tumor-derived exosomes orchestrate mast cell activation to foster a pro-metastatic niche. Specifically, exosomes derived from GC cells and tumor tissues are rich in stem cell factors (SCF) compared to normal gastric epithelial cells and gastric tissues. These exosomes deliver SCF to mast cells, activating the c-KIT receptor and triggering degranulation. The subsequent release of tryptase from activated mast cells promotes angiogenesis by activating the MAPK pathway in endothelial cells and upregulating VEGF expression. This SCF/c-KIT/tryptase axis was validated as critical for promoting metastasis in a mouse model. Our findings position tumor-derived exosomes as central regulators that bridge GC cells with mast cells to promote angiogenesis and tumor metastasis.

The established paradigm posits that mast cell infiltration and activation within the tumor microenvironment are primarily driven by the interaction between tumor-derived SCF and the c-KIT receptor on mast cells [14]. But the precise mechanism through which tumor cells deliver SCF to these immune cells has remained elusive [14]. Our study provides novel insight into this process, identifying the exosomal pathway as a critical mediator in the tumor-mediated activation of mast cells. We observed that the SCF content within exosomes secreted by gastric cancer (GC) cells was significantly elevated compared to that derived from normal gastric epithelial cells. Functionally, these GC-derived exosomes were capable of promoting both the degranulation and chemotactic recruitment of mast cells. These findings collectively indicate that exosomes might serve as an important vehicle for the transfer of bioactive SCF, thereby facilitating a direct functional crosstalk between GC cells and mast cells. From a therapeutic perspective, this dependency on exosomal SCF delivery suggests that interfering with exosome biogenesis, release, or uptake could represent a novel strategy to block mast cell activation and subsequent metastasis. Pharmacological inhibitors of exosome production (e.g., GW4869, an inhibitor of neutral sphingomyelinase-2) have shown efficacy in preclinical cancer models and could be repurposed to disrupt this axis [15]. This role of tumor exosomes in modulating mast cell function is corroborated by emerging evidence from other malignancies. For instance, Ben et al. demonstrated that exosomes from lung cancer cells can induce mast cell degranulation and alter their cytokine secretion profile, contributing to cancer-associated coagulation disorders [16]. Similarly, in pancreatic ductal adenocarcinoma, tumor-derived exosomes were shown to deliver miR-188-5p to tumor-associated mast cells. This microRNA stabilizes the transcription factor ERG by inhibiting its ubiquitin-mediated degradation, consequently enhancing ERG-driven transcription of pro-tumorigenic factors like CXCL10 [17]. Together with our data, these studies underscore a conserved mechanism across cancer types, wherein exosomes function as a key communication medium, enabling tumor cells to remotely reprogram mast cell activity within the tumor and pre-metastatic niches. Therefore, targeting the shared exosomal machinery or the specific cargo–receptor interactions (such as SCF/c-KIT) might yield broad-spectrum anti-metastatic therapies and warrants preclinical and ultimately clinical evaluation.

Our findings further delineate a specific pro-angiogenic mechanism initiated by exosome-activated mast cells, namely through the secretion of tryptase. As a major serine protease stored in mast cell granules, tryptase is a well-characterized and potent mediator of angiogenesis [18]. In the context of GC, clinical correlative studies support this function. For instance, Ammendola et al. reported a significant positive correlation between tryptase expression and microvessel density in both primary GC tissues and metastatic lymph nodes [19]. The same group also identified a positive association between tryptase-positive mast cells within tumors and the number of metastatic lymph nodes [20]. While these clinical correlations have been established through histological analyses, definitive experimental evidence directly linking mast cell-derived tryptase to the stimulation of new blood vessel formation has been lacking. Our study provides this causal evidence. By employing mast cells with stable knockdown of tryptase, we demonstrated that their conditioned medium lost the capacity to induce endothelial tube formation or activate the MAPK signaling pathway in HUVECs. This functional ablation experiment positions mast cell-derived tryptase as a critical downstream effector molecule within the exosome-mast cell communication axis. It is not merely a correlative marker but is directly responsible for executing the pro-angiogenic program that establishes the vascular network essential for supporting metastatic growth. Thus, our work translates previous observational correlations into a defined mechanistic pathway. Importantly, tryptase itself emerges as a druggable target. Specific tryptase inhibitors (such as nafamostat mesylate) have been developed for allergic and inflammatory diseases. Moreover, research results indicate that their potential repurposing to suppress tumor angiogenesis and metastasis in gastric cancer [21]. Similarly, the downstream MAPK pathway and VEGF are clinically validated anti-cancer targets. Combining conventional VEGF inhibitors (e.g., bevacizumab) with MAPK pathway blockers (e.g., trametinib) may augment the anti-angiogenic effect by simultaneously disrupting the mast cell-derived pro-angiogenic signal [22].

Tumor-derived exosomes serve as critical mediators in the metastatic cascade by transferring bioactive molecules, including proteins, lipids, and nucleic acids, to recipient stromal cells within the tumor microenvironment, such as fibroblasts and various immune cell populations, thereby facilitating metastatic progression [23]. In the present study, our *in vivo* findings demonstrate that exosomes isolated from gastric tumor tissues markedly promote the formation of lung metastatic foci. This pro-metastatic effect was associated with the recruitment and aggregation of mast cells at pre-metastatic sites, as well as enhanced angiogenesis. Significantly, administration of a c-KIT blocking antibody effectively abolished these exosome-driven effects, suggesting the functional importance of this signaling axis. While inhibitors targeting the SCF/c-KIT pathway are established therapeutics in malignancies like gastrointestinal stromal tumors, melanoma, and acute myeloid leukemia [24], their potential utility in GC is not well defined. Supporting a role for this pathway in therapeutic resistance, Su et al. reported that the tyrosine kinase inhibitors imatinib and apatinib restored pyrotinib sensitivity in resistant models by suppressing SCF/c-KIT signaling [25]. Our *in vivo* data using a c-KIT-blocking antibody provide direct evidence that interrupting this specific receptor-ligand interaction can suppress exosome-induced lung metastasis and angiogenesis. Beyond antibodies, small-molecule tyrosine kinase inhibitors that target c-KIT, such as imatinib [26], sunitinib [27], and regorafenib [28], are clinically available and may merit future investigation in gastric cancer patients with high exosomal SCF or activated mast cell signatures. Collectively, our data propose that intercepting the communication between tumor exosomes and mast cells, specifically by inhibiting the SCF/c-KIT interaction, represents a promising therapeutic strategy to disrupt the formation of a pro-metastatic niche in GC after pending future validation in patient cohorts.

There were still some limitations that required attention. First, while the LAD2 cell line is a standard human mast cell model, its behavior may not fully recapitulate that of tissue-resident mast cell subsets. Future studies should validate key findings in primary human mast cells. Second, the mouse metastasis model, though informative, does not mirror the complex, multi-step progression of spontaneous GC metastasis. Genetic mouse models of GC could provide complementary insights. Thirdly, the main focus of this study was on the interaction between GC tumor cells and mast cells. Other immune cells and stromal cells, such as fibroblasts, also play a role in the TME and tumor metastasis. The influence of exosome-derived SCF on them needs to be further clarified through future research. Fourthly, although the sample size of 5 mice per group was based on a power analysis, it was relatively small in the transfer study. This sample size may not adequately reflect the differences among individuals, and thus, the results should be interpreted with caution. It is necessary to conduct larger-scale studies in the future to verify the robustness of the observed transfer effect. Finally, all the findings of this study were derived from cell lines, culture media, exosome formulations, and mouse models. No tests were conducted on human gastric cancer tissues, patient-derived plasma exosomes, or mast cell infiltration. Therefore, we were unable to assess whether the levels of exosome SCF or the activation state of mast cells were related to clinical outcomes such as metastasis, recurrence, or patient survival. Although our mechanism studies suggested potential translational application directions, such as the SCF/c-KIT/trypsin axis as a therapeutic target, or exosome SCF as a biomarker for liquid biopsy, these hypotheses remain speculative and require rigorous validation in population cohorts. Despite these limitations, the multiple druggable nodes we have identified (exosome biogenesis, SCF/c-KIT, tryptase, MAPK, VEGF) provide a preliminary rationale for exploring combination therapies that target both the exosome–mast cell axis and conventional pro-angiogenic pathways. However, further preclinical and clinical validation is required before any therapeutic application can be considered.

## 5 Conclusion

In summary, we have identified a coherent signaling circuit in GC: tumor-derived exosomes deliver SCF to activate mast cells via c-KIT, leading to tryptase-dependent angiogenesis and metastasis promotion. This work illuminates exosomes as critical mediators of tumor-stroma crosstalk and establishes the SCF/c-KIT axis on mast cells as a potential therapeutic target to disrupt the metastatic microenvironment in gastric cancer. Furthermore, our findings highlight several actionable molecular and immunological determinants for future drug development. These include inhibition of exosome release, blockade of SCF/c-KIT interaction with neutralizing antibodies or small-molecule kinase inhibitors, neutralization of mast cell-derived tryptase, and disruption of downstream MAPK/VEGF signaling. The successful clinical application of c-KIT inhibitors in other malignancies, together with our *in vivo* proof-of-concept using a c-KIT blocking antibody, strongly supports the repurposing of these agents for gastric cancer patients with an activated exosome–mast cell axis. Moving forward, combination regimens that simultaneously target exosomal SCF/c-KIT signaling and other pro-angiogenic pathways may offer enhanced anti-metastatic efficacy. Ultimately, the molecular determinants dissected in this study are not merely pathogenic mediators but represent a rich repertoire of therapeutic vulnerabilities for future precision oncology strategies in gastric cancer.

## Data Availability

The data that support the findings of this study are available from the Corresponding Author, [Kai Jia], upon reasonable request.

## Abbreviations

DMEM	Dulbecco’s Modified Eagle Medium
ECM	Endothelial Cell Medium
ELISA	Enzyme-Linked Immunosorbent Assay
FBS	Fetal Bovine Serum
GC	Gastric Cancer
NTA	Nanoparticle Tracking Analysis
PS	Penicillin-Streptomycin
qRT-PCR	Quantitative Real-Time PCR
SCF	Stem Cell Factor
TEM	Transmission Electron Microscope
TME	Tumor Microenvironment
IHC	Immunohistochemistry
IF	Immunofluorescence
Exo	Exosome
NTA	Nanoparticle Tracking Analysis
IVIS	*In Vivo* Imaging System
H&E	Hematoxylin and Eosin

## References

[1] Bray F , Laversanne M , Sung H , Ferlay J , Siegel RL , Soerjomataram I , et al. Global cancer statistics 2022: GLOBOCAN estimates of incidence and mortality worldwide for 36 cancers in 185 countries. CA Cancer J Clin. 2024; 74( 3): 229– 63. doi:10.3322/caac.21834. 38572751

[2] Duan Y , Xu Y , Dou Y , Xu D . *Helicobacter pylori* and gastric cancer: Mechanisms and new perspectives. J Hematol Oncol. 2025; 18( 1): 10. doi:10.1186/s13045-024-01654-2. PMC11756206 39849657

[3] Yasuda T , Wang YA . Gastric cancer immunosuppressive microenvironment heterogeneity: Implications for therapy development. Trends Cancer. 2024; 10( 7): 627– 42. doi:10.1016/j.trecan.2024.03.008. PMC11292672 38600020

[4] Li P , Zhang H , Chen T , Zhou Y , Yang J , Zhou J . Cancer-associated fibroblasts promote proliferation, angiogenesis, metastasis and immunosuppression in gastric cancer. Matrix Biol. 2024; 132: 59– 71. doi:10.1016/j.matbio.2024.06.004. 38936680

[5] Sammarco G , Varricchi G , Ferraro V , Ammendola M , de Fazio M , Altomare DF , et al. Mast cells, angiogenesis and lymphangiogenesis in human gastric cancer. Int J Mol Sci. 2019; 20( 9): 2106. doi:10.3390/ijms20092106. PMC6540185 31035644

[6] Guidolin D , Ruggieri S , Annese T , Tortorella C , Marzullo A , Ribatti D . Spatial distribution of mast cells around vessels and glands in human gastric carcinoma. Clin Exp Med. 2017; 17( 4): 531– 9. doi:10.1007/s10238-017-0452-7. 28105541

[7] Tang XH , Guo T , Gao XY , Wu XL , Xing XF , Ji JF , et al. Exosome-derived noncoding RNAs in gastric cancer: Functions and clinical applications. Mol Cancer. 2021; 20( 1): 99. doi:10.1186/s12943-021-01396-6. PMC8323226 34330299

[8] He C , Zhou Z , Ye J , Chen X , Yang Y , Jin X , et al. *TGF-βI*/*FERMT2*/*COL6A1* reciprocal loop drives tumor-stroma crosstalk and promotes peritoneal metastasis in gastric cancer. Int J Biol Sci. 2025; 21( 13): 5859– 73. doi:10.7150/ijbs.119895. PMC12509910 41079932

[9] Li W , Wei H , Liu J , Zhao Z , Wang F , Qiao L , et al. Exosomal Biglycan promotes gastric cancer progression via M2 polarization and CXCL10-mediated JAK/STAT1 activation. Cancer Lett. 2025; 626: 217758. doi:10.1016/j.canlet.2025.217758. 40311912

[10] Tsai M , Valent P , Galli SJ . KIT as a master regulator of the mast cell lineage. J Allergy Clin Immunol. 2022; 149( 6): 1845– 54. doi:10.1016/j.jaci.2022.04.012. PMC9177781 35469840

[11] Sheikh E , Tran T , Vranic S , Levy A , Bonfil RD . Role and significance of c-KIT receptor tyrosine kinase in cancer: A review. Bosn J Basic Med Sci. 2022; 22( 5): 683– 98. doi:10.17305/bjbms.2021.7399. PMC9519160 35490363

[12] Roy LD , Curry JM , Sahraei M , Besmer DM , Kidiyoor A , Gruber HE , et al. Arthritis augments breast cancer metastasis: Role of mast cells and SCF/c-Kit signaling. Breast Cancer Res. 2013; 15( 2): R32. doi:10.1186/bcr3412. PMC3672823 23577751

[13] Kirshenbaum AS , Akin C , Wu Y , Rottem M , Goff JP , Beaven MA , et al. Characterization of novel stem cell factor responsive human mast cell lines LAD 1 and 2 established from a patient with mast cell sarcoma/leukemia; activation following aggregation of FcepsilonRI or FcgammaRI. Leuk Res. 2003; 27( 8): 677– 82. doi:10.1016/s0145-2126(02)00343-0. 12801524

[14] Huang B , Lei Z , Zhang GM , Li D , Song C , Li B , et al. SCF-mediated mast cell infiltration and activation exacerbate the inflammation and immunosuppression in tumor microenvironment. Blood. 2008; 112( 4): 1269– 79. doi:10.1182/blood-2008-03-147033. PMC2515142 18524989

[15] Zhu X , Li T , Wang Q , Yan K , Ma S , Lin Y , et al. Dual-synergistic nanomodulator alleviates exosomal PD-L1 expression enabling exhausted cytotoxic T lymphocytes rejuvenation for potentiated iRFA-treated hepatocellular carcinoma immunotherapy. ACS Nano. 2024; 18( 47): 32818– 33. doi:10.1021/acsnano.4c11257. 39528907

[16] Ben S , Huang X , Shi Y , Xu Z , Xiao H . Change in cytokine profiles released by mast cells mediated by lung cancer-derived exosome activation may contribute to cancer-associated coagulation disorders. Cell Commun Signal. 2023; 21( 1): 97. doi:10.1186/s12964-023-01110-7. PMC10161433 37143160

[17] Yin H , Chen Q , Gao S , Shoucair S , Xie Y , Habib JR , et al. The crosstalk with CXCL10-rich tumor-associated mast cells fuels pancreatic cancer progression and immune escape. Adv Sci. 2025; 12( 14): e2417724. doi:10.1002/advs.202417724. PMC11984875 39965084

[18] Longo V , Tamma R , Brunetti O , Pisconti S , Argentiero A , Silvestris N , et al. Mast cells and angiogenesis in pancreatic ductal adenocarcinoma. Clin Exp Med. 2018; 18( 3): 319– 23. doi:10.1007/s10238-018-0493-6. 29492715

[19] Ammendola M , Sacco R , Zuccalà V , Luposella M , Patruno R , Gadaleta P , et al. Mast cells density positive to tryptase correlate with microvascular density in both primary gastric cancer tissue and loco-regional lymph node metastases from patients that have undergone radical surgery. Int J Mol Sci. 2016; 17( 11): 1905. doi:10.3390/ijms17111905. PMC5133903 27854307

[20] Ammendola M , Sacco R , Donato G , Zuccalà V , Russo E , Luposella M , et al. Mast cell positivity to tryptase correlates with metastatic lymph nodes in gastrointestinal cancer patients treated surgically. Oncology. 2013; 85( 2): 111– 6. doi:10.1159/000351145. 23887206

[21] Leporini C , Ammendola M , Marech I , Sammarco G , Sacco R , Gadaleta CD , et al. Targeting mast cells in gastric cancer with special reference to bone metastases. World J Gastroenterol. 2015; 21( 37): 10493– 501. doi:10.3748/wjg.v21.i37.10493. PMC4588072 26457010

[22] Hurley-Novatny AC , Allbritton-King JD , Jha S , Cowen EW , Colbert RA , Navid F , et al. Fibroblasts from patients with melorheostosis promote angiogenesis in healthy endothelial cells through secreted factors. J Investig Dermatol. 2022; 142( 9): 2406– 14.e5. doi:10.1016/j.jid.2022.02.006. PMC9388700 35189151

[23] Mashouri L , Yousefi H , Aref AR , Ahadi AM , Molaei F , Alahari SK . Exosomes: Composition, biogenesis, and mechanisms in cancer metastasis and drug resistance. Mol Cancer. 2019; 18( 1): 75. doi:10.1186/s12943-019-0991-5. PMC6444571 30940145

[24] Pathania S , Pentikäinen OT , Singh PK . A holistic view on c-Kit in cancer: Structure, signaling, pathophysiology and its inhibitors. Biochim Biophys Acta Rev Cancer. 2021; 1876( 2): 188631. doi:10.1016/j.bbcan.2021.188631. 34606974

[25] Su B , Huang T , Jin Y , Yin H , Qiu H , Yuan X . Apatinib exhibits synergistic effect with pyrotinib and reverses acquired pyrotinib resistance in HER2-positive gastric cancer via stem cell factor/c-kit signaling and its downstream pathways. Gastric Cancer. 2021; 24( 2): 352– 67. doi:10.1007/s10120-020-01126-9. PMC7902570 33030616

[26] Bertsimas D , Margonis GA , Sujichantararat S , Koulouras A , Ma Y , Antonescu CR , et al. Interpretable artificial intelligence to optimise use of imatinib after resection in patients with localised gastrointestinal stromal tumours: An observational cohort study. Lancet Oncol. 2024; 25( 8): 1025– 37. doi:10.1016/S1470-2045(24)00259-6. PMC12051465 38976997

[27] Kawabata K , Takahashi T , Nishida T , Kurokawa Y , Yamamoto K , Saito T , et al. Clinical impact of primary and secondary KIT mutations on the efficacy of molecular-targeted therapies in gastrointestinal stromal tumors. Gastric Cancer. 2025; 28( 5): 899– 910. doi:10.1007/s10120-025-01639-1. PMC12378141 40699464

[28] Cousin S , Guégan JP , Shitara K , Palmieri LJ , Metges JP , Pernot S , et al. Identification of microenvironment features associated with primary resistance to anti-PD-1/PD-L1 + antiangiogenesis in gastric cancer through spatial transcriptomics and plasma proteomics. Mol Cancer. 2024; 23( 1): 197. doi:10.1186/s12943-024-02092-x. PMC11396962 39272096

